# Naturally-Derived Amphiphilic Polystyrenes Prepared by Aqueous Controlled/Living Cationic Polymerization and Copolymerization of Vinylguaiacol with R–OH/BF_3_·OEt_2_

**DOI:** 10.3390/polym10121404

**Published:** 2018-12-18

**Authors:** Hisaaki Takeshima, Kotaro Satoh, Masami Kamigaito

**Affiliations:** Department of Molecular and Macromolecular Chemistry, Graduate School of Engineering, Nagoya University Furo-cho, Chikusa-ku, Nagoya 464-8603, Japan; takeshima@chiral.apchem.nagoya-u.ac.jp

**Keywords:** cationic polymerization, controlled/living polymerization, aqueous media, naturally-occurring monomer, phenolic styrene, amphiphilic polymer

## Abstract

In this study, we investigated direct-controlled/living cationic polymerization and copolymerization of 4-vinylguaiacol (4VG), i.e., 4-hydroxy-3-methoxystyrene, which can be derived from naturally-occurring ferulic acid, to develop novel bio-based amphiphilic polystyrenes with phenol functions. The controlled/living cationic polymerization of 4VG was achieved using the R–OH/BF_3_·OEt_2_ initiating system, which is effective for the controlled/living polymerization of petroleum-derived 4-vinylphenol in the presence of a large amount of water via reversible activation of terminal C–OH bond catalyzed by BF_3_·OEt_2_, to result in the polymers with controlled molecular weights and narrow molecular weight distributions. The random or block copolymerization of 4VG was also examined using *p*-methoxystyrene (pMOS) as a comonomer with an aqueous initiating system to tune the amphiphilic nature of the 4VG-derived phenolic polymers. The obtained polymer can be expected not only to be used as a novel styrenic bio-based polymer but also as a material with amphiphilic nature for some applications.

## 1. Introduction

Living or controlled polymerization is one of the most facile methods for preparing various well-defined macromolecular architectures with functional groups, including amphiphilic block copolymers that consist both of hydrophilic and hydrophobic segments [1]. The living cationic polymerization has been conducted using a binary initiating system containing a protonic acid or its adduct of monomer (initiator) and a metal-based Lewis acid (co-initiator or catalyst), which has also been applied for the synthesis of a series of amphiphilic polymers that consist of functional vinyl ether or styrene [2,3,4,5,6,7]. In cationic polymerization, however, the reaction is generally performed in a rigorously-dried medium. This is because a small amount of water could be an initiator, namely, a so-called “cationogen”, when coupled with a strong Lewis acid. In large amounts, water can deactivate the catalyst or growing cation via hydrolysis and often induces chain-transfer reactions to produce only low-molecular-weight polymers. In contrast, approximately 20 years ago, controlled/living cationic polymerization in a water system was realized with BF_3_·OEt_2_ as the Lewis acid, which possibly induced selective activation of a stable carbon-oxygen bond (R–OH) that was generated by the reaction between the carbocation and water [8,9,10,11,12,13,14]. Furthermore, the R–OH/BF_3_·OEt_2_ system was applicable even for the direct controlled/living cationic polymerization of *p*-hydroxystyrene (pHS) or 4-vinylphenol without any protective groups to produce functional hydrophilic polymers that have a phenolic group in the side chain and a controlled molecular weight.

From the viewpoint of sustainable development, in recent years, the use of plant-derived compounds has become important [15,16,17,18,19,20,21,22,23,24,25,26,27,28,29,30,31]. We have also conducted polymerization studies of various naturally-derived vinyl compounds as raw materials for creating novel bio-based polymers [32,33,34,35,36,37,38,39,40,41,42,43,44,45,46,47,48,49]. One of these has a phenylpropanoid skeleton, which mainly occurs in vegetable oil, such as β-methylstyrenes and cinnamic acids [50,51,52]. These compounds are biosynthesized in plant cells, especially for the production of lignin via oxidative coupling or cross-linking [53,54]. We found that β-methylstyrene of isoeugenol, which is considered to be an amphiphilic monomer that has a phenolic hydroxyl and a hydrophobic methoxy group in the pendant group, did not homopolymerize, but could be copolymerized with *p*-methoxystyrene (pMOS) by the aqueous R–OH/BF_3_·OEt_2_ system [44]. Although the copolymerization of isoeugenol produced copolymers that contain naturally-derived phenolic groups, the hydrophilicity could not be changed due to the alternating cross-propagation during the copolymerization with pMOS.

A similar amphiphilic styrene to isoeugenol has been found among natural products, which is 4-hydroxy-3-methoxystyrene [55,56,57,58], a naturally-occurring fragrance component called 4-vinylguaiacol (4VG). This compound can also be prepared from the naturally-abundant ferulic acid via decarboxylation [59,60]. Recently, we also reported that the controlled radical homopolymerization of protected 4VG successfully proceeded via reversible addition-fragmentation chain transfer (RAFT) polymerization or nitroxide-mediated polymerization (NMP), where the phenolic functional group had to be protected even for radical polymerization because the phenolic group generally acts as an inhibitor or retarder in radical reactions [47].

**Scheme 1:**
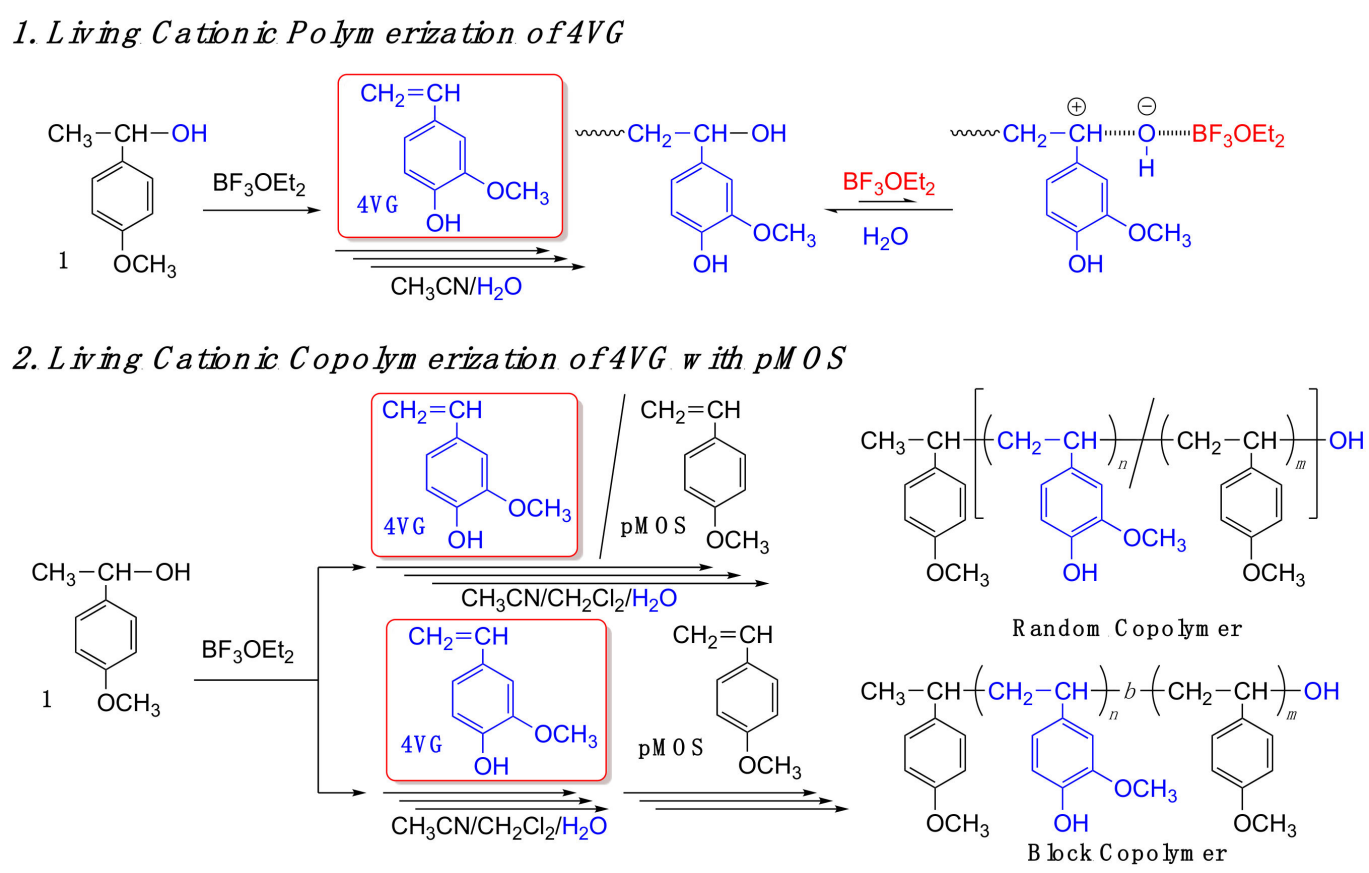
Synthesis of bio-based amphiphilic polystyrenes from 4-vinylguaiacol (4VG) using aqueous-controlled/living-cationic polymerization and copolymerization.

In this study, we aimed to develop novel amphiphilic-phenolic bio-based polystyrenes by direct homopolymerization and copolymerization of 4VG without using any protective groups. In particular, the direct-cationic homopolymerization of 4VG was examined in an aqueous system using the R–OH/BF_3_·OEt_2_ system and copolymerization with pMOS to produce statistical and block copolymers (Scheme 1). The properties of the various types of 4VG-based polystyrenes obtained were also evaluated for use as naturally-derived amphiphilic polystyrenes. This study thus enables the direct synthesis of novel bio-based amphiphilic polystyrenes with phenolic functions without using tedious protection and deprotection procedures, which will be a great advantage in terms of not only renewable functional materials but also facile synthetic procedures.

## 2. Materials and Methods

### 2.1. Materials

4-Vinylguaiacol (4VG) (Aldrich, St. Louis, MO, USA, >98%) and *p*-methoxystyrene (pMOS) (4-vinylanisole; Aldrich; 97%) were distilled under reduced pressure before use. The water adduct of pMOS (**1**: 4-methoxy-α-methylbenzyl alcohol (Aldrich; 99%)) was distilled over calcium hydride (2 mmHg, 110 °C) before use. BF_3_·OEt_2_ (Aldrich; purified, redistilled) and acetonitrile (CH_3_CN) (FUJIFILM Wako Pure Chemical Corporation, Osaka, Japan, H_2_O < 50 ppm) were used as received. Dichloromethane (CH_2_Cl_2_) (KANTO, Tokyo, Japan, >99.5%; H_2_O < 0.005%) was deoxygenized and dried by passing through a column from Glass Contour Systems (Glass Contour, Nashua, NH, USA) before use. Distilled deionized water was used for the polymerizations without degassing. Acetic anhydride (TCI, Tokyo, Japan, >99%) and pyridine (TCI, >99%) were used as received.

### 2.2. Aqueous Cationic Polymerization of 4-vinylguaiacol (4VG)

The polymerization of 4VG was performed by the syringe technique under dry nitrogen in baked glass tubes equipped with a three-way stopcock. A typical example for 4VG polymerization is given below. The polymerization was initiated by adding solutions of BF_3_·OEt_2_ (20 mM in CH_3_CN; 0.50 mL) into a monomer solution (4.5 mL) that contained 4VG (0.14 mL, 1.0 mmol) and **1** (2.8 μL, 0.02 mmol), and water (0.018 mL) and *o*-C_6_H_4_Cl_2_ as an internal standard (0.08 mL) in CH_3_CN (4.27 mL) at 0 °C. The total volume of the reaction mixture was thus 5.0 mL. After a predetermined time, the polymerization was terminated by diluting with methanol (1.0 mL). Monomer conversion was determined from the concentration of the residual monomers as measured by ^1^H NMR (95% for 1 h). The polymer was isolated via precipitation into a mixture of *n*-hexane and toluene (1:1; 100 mL). The filtrated precipitate was dissolved in methanol and evaporated until dry under a reduced pressure to produce the polymer product (*M*_n_ = 12,800, *M*_w_/*M*_n_ = 1.37).

### 2.3. Block Copolymerization of 4VG and pMOS with the R–OH/BF_3_·OEt_2_ System

The block copolymerization of 4VG and pMOS was performed via the syringe technique under dry nitrogen in baked glass tubes equipped with a three-way stopcock. A typical example of the block copolymerization is given below. The polymerization of 4VG was first initiated by adding solutions of BF_3_·OEt_2_ (20 mM in CH_3_CN; 0.50 mL) into a monomer solution (4.5 mL) that contained 4VG (0.068 mL, 0.50 mmol), 1 (2.8 μL, 0.02 mmol), water (0.018 mL), and *o*-C_6_H_4_Cl_2_ as an internal standard (0.07 mL) in the mixture of CH_3_CN (3.34 mL) and CH_2_Cl_2_ (1.0 mL) at 0 °C. The total volume of the reaction mixture was 5.0 mL. After 1 h (the conversion of 4VG = 88%, *M*_n_ = 7000, *M*_w_/*M*_n_ = 1.32), pMOS (0.067 mL, 0.50 mmol) was added into the reaction solution. After 40 h, the polymerization was terminated by diluting with methanol (1.0 mL). The conversions of 4VG and pMOS were determined from the concentrations of the residual monomers as measured by ^1^H NMR (4VG: >99% and pMOS: 95%). The solution was diluted with ethyl acetate (150 mL), washed with water to remove initiator residues, and evaporated until dry under reduced pressure to give the block copolymer product (*M*_n_ = 11,100, *M*_w_/*M*_n_ = 1.42, 4VG/pMOS = 51/49).

### 2.4. Measurements

^1^H NMR spectra were recorded on a JEOL ECS-400 spectrometer (JEOL, Tokyo, Japan) operated at 400 MHz. The number-average molecular weights (*M*_n_) and molecular weight distributions (*M*_w_/*M*_n_) of the product polymers were determined via size-exclusion chromatography (SEC) in DMF containing 100 mM LiCl at 40 °C on two hydrophilic polymer gel columns [Tosoh α-M + α-3000 (7.8 mm i.d. × 30 cm) (Tosoh Corporation, Tokyo, Japan); flow rate of 1.0 mL/min] (for poly(4VG)) connected to a JASCO PU-2080 precision pump and a JASCO RI-2031 detector (JASCO, Tokyo, Japan). The columns were calibrated against standard polystyrene samples (Agilent Technologies, Santa Clara, CA, USA; *M*_p_ = 580–3,053,000, *M*_w_/*M*_n_ = 1.02–1.23). The glass transition temperature (*T*_g_) of the polymers was recorded on a Q200 differential-scanning calorimeter (TA Instruments, Inc., New Castle, DE, USA), and the *T*_g_ values were obtained from the second scan after removing the thermal history. The samples were first heated to 210 °C at 10 °C/min, equilibrated at this temperature for 10 min, and cooled to −60 °C at 5 °C/min. After holding at this temperature for 5 min, the samples were then reheated to 210 or 260 °C at 10 °C/min. The thermogravimetric analyses (TGA) were performed on a Q500 system (TA Instruments Inc.) at 5 °C/min under a N_2_ gas flow.

## 3. Results and Discussion

### 3.1. Poly(vinylguaiacol) via Aqueous Cationic Polymerization

**Figure 1:**
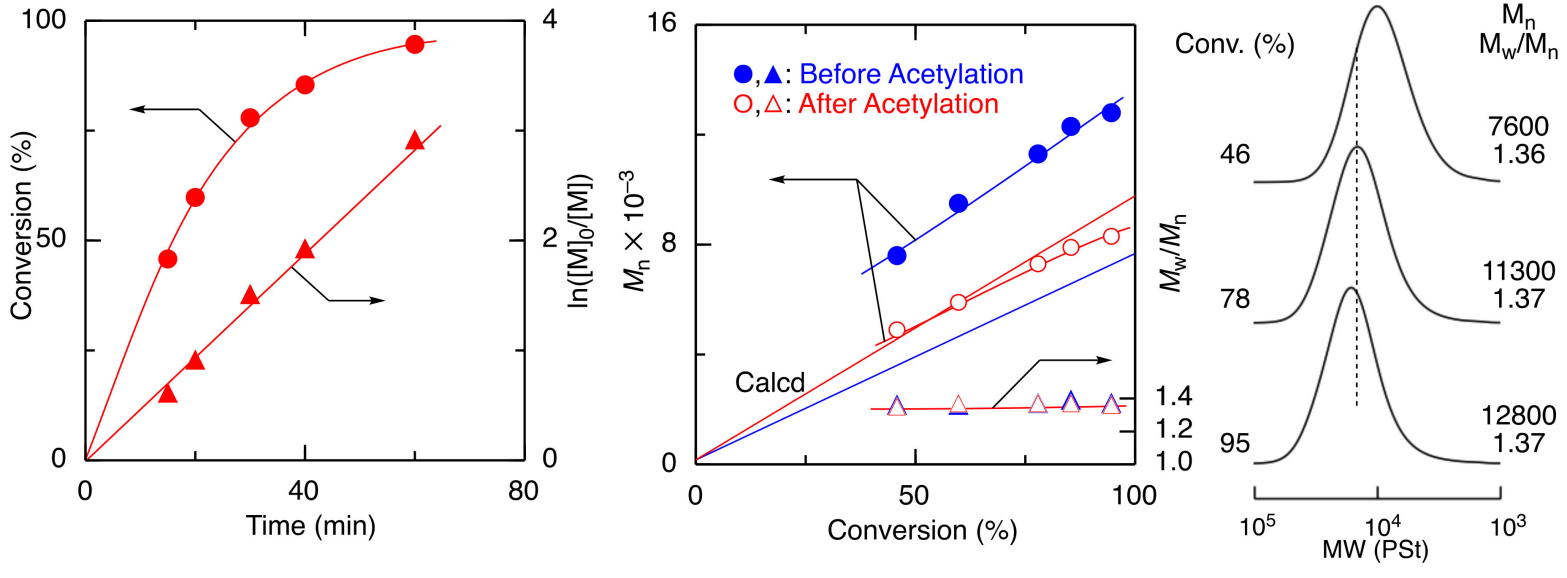
Aqueous-controlled/living-cationic polymerization of 4VG in CH_3_CN at 0 °C; [4VG]_0_/[**1**]_0_/[BF_3_·OEt_2_]_0_/[H_2_O]_0_ = 200/4.0/2.0/200 mM. For *M*_n_ and *M*_w_/*M*_n_ curves, the diagonal line indicates the calculated *M*_n_ assuming the formation of one polymer chain per **1** molecule.

The direct cationic polymerization of the bio-based phenolic monomer, 4VG, was investigated in an aqueous solution of CH_3_CN using an initiating system consisting of the water adduct of pMOS (**1**) as the initiator and BF_3_·OEt_2_ as the Lewis acid catalyst, which was effective for controlling the polymerization of pHS as reported in previous literature [8]. The polymerization of 4VG was performed in CH_3_CN/H_2_O at 0 °C in which the concentration of water was equal to that of the monomer ([H_2_O]_0_ = [4VG]_0_ = 200 mM) with large excess of catalyst ([BF_3_·OEt_2_]_0_ = 2.0 mM). As shown in Figure 1, the 4VG polymerization proceeded smoothly, was of first-order with respect to monomer, and reached complete conversion (95%) in 1 h, indicating that the catalyst was not decomposed or deactivated by either the added water or phenolic monomer. The polymerization of 4VG was faster than that of pHS (95% conversion in 3 h) under the same condition, suggesting a higher reactivity of 4VG due to the two electron-donating substituents on the phenyl group. The SEC curves of the obtained polymer were unimodal and shifted as the polymerization proceeded, maintaining a relatively narrow molecular weight distribution (MWD) (*M*_w_/*M*_n_ ~ 1.3). The *M*_n_ increased linearly but was higher than the calculated value, assuming that one polymer chain is formed from one initiator **1** molecule, which was likely due to the difference in hydrodynamic volume compared with standard polystyrene. Then, the acetylation of phenolic groups in the obtained polymer was achieved using an excess amount of acetic anhydride at 100 °C in pyridine [8]. As a result, quantitative acetylation proceeded to result in an *M*_n_ for the polymer close to the calculated value at each conversion. A slightly higher *M*_n_ at a low monomer conversion was most probably due to the loss of low-molecular weight polymers during recovery of the polymers by precipitation. Thus, the aqueous-controlled/living-cationic polymerization of 4VG likely proceeded to form one molecule of polymer from one initiator molecule as in the case of the pHS polymerization.

**Figure 2:**
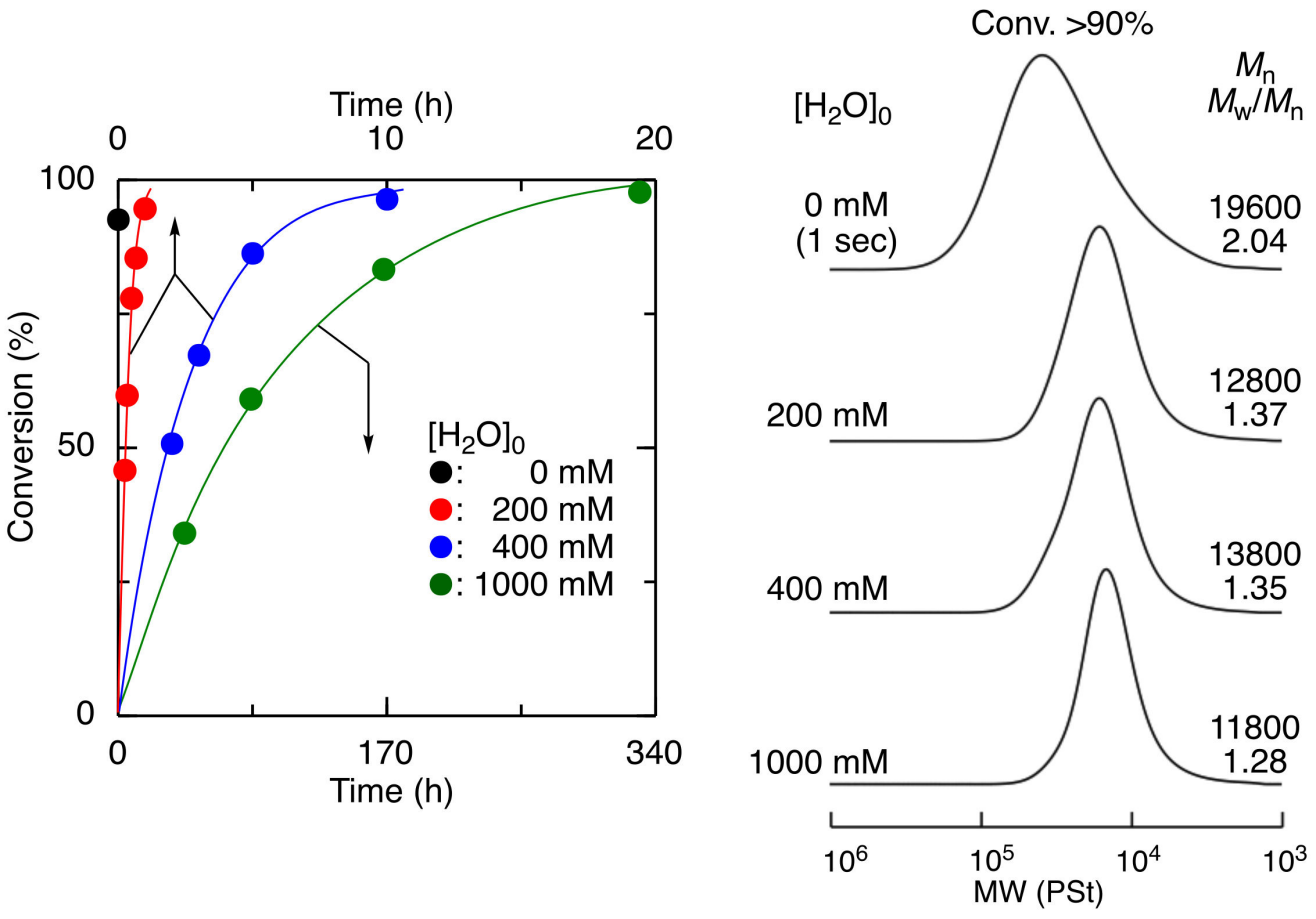
Effects of water on the controlled/living cationic polymerization of 4VG in CH_3_CN at 0 °C; [4VG]_0_/[pMOS-OH]_0_/[BF_3_·OEt_2_]_0_/[H_2_O]_0_ = 200/4.0/2.0/0, 200, 400, or 1000 mM.

This reaction system requires added water not for initiation but for polymerization control, whereas water usually acts as a chain-transfer agent or initiator (cationogen) in conventional cationic polymerization. Therefore, the effect of added water was also investigated for the cationic polymerization of 4VG in which the polymerization was performed at various water concentrations, i.e., from that equivalent to the monomer (200 mM) to excess amounts (400 mM and 1.0 M) (Figure 2). Although the addition of water clearly retarded the polymerization, the polymerization actually proceeded without prohibition even in the presence of a large excess of water. In addition, the MWD of the produced polymer became narrower as the amount of added water increased, whereas the *M*_n_ was not affected by the water concentration. This finding further indicates that the reaction of water with carbocations does not induce either chain-transfer or a termination reaction in this system but biases the equilibrium at the growing end between the alcohol and cationic species toward the dormant species.

**Figure 3:**
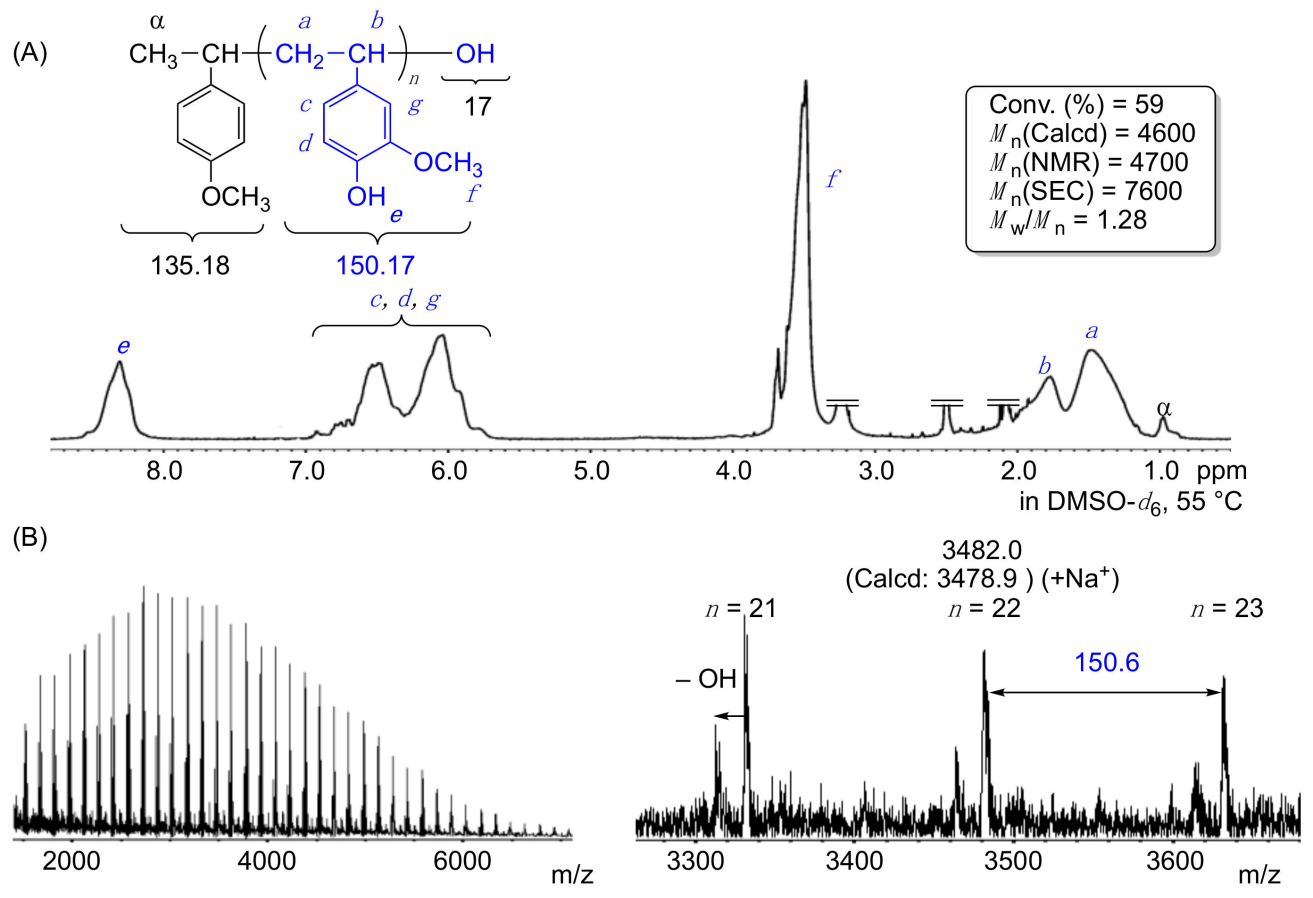
^1^H NMR (**A**) and MALDI-TOF-MS (**B**) spectrum of poly(4VG) obtained with **1**/BF_3_·OEt_2_ in CH_3_CN at 0 °C in the presence of water; [4VG]_0_/[**1**]_0_/[BF_3_·OEt_2_]_0_/[H_2_O]_0_ = 200/4.0/2.0/1000 mM.

The polymer thus obtained from 4VG was analyzed by ^1^H NMR and MALDI-TOF-MS. In the ^1^H NMR spectrum (Figure 3A), a peak for the unprotected pendent hydroxyl group (*e*) was observed in addition to peaks derived from the main chain (*a* and *b*), aromatic group (*c*, *d*, and *g*), and pendent methoxy group (*f*), indicating that the 4VG polymer was produced by addition polymerization of the vinyl group. Furthermore, the molecular weight calculated from the integral ratios of peak α derived from initiator **1** and peak *f* of the methoxy group [*M*_n_(NMR) = 4700] agreed well with the calculated value obtained from the monomer conversion [*M*_n_(calcd) = 4600]. The MALDI-TOF-MS spectrum exhibited peaks separated by a molecular weight interval for 4VG in which the absolute molecular weight of each main peak was consistent with the polymers initiated with the methoxystyrene unit and almost terminated with the hydroxyl group (Figure 3B). These results also support that controlled/living polymers with a well-controlled terminal structure were obtained by aqueous-cationic polymerization of 4VG without any protecting groups.

### 3.2. Statistical and Block Copolymers of Vinylguaiacol with p-Methoxystyrene

Based on the success of the direct-controlled/living cationic polymerization of 4VG in the aqueous system, we then studied random/statistical and block copolymerization of 4VG. Herein, pMOS was employed as the comonomer, which is a cationically polymerizable and more hydrophobic styrene derived from petrochemicals, to prepare various types of 4VG-derived phenolic polystyrene copolymers [9].

**Figure 4:**
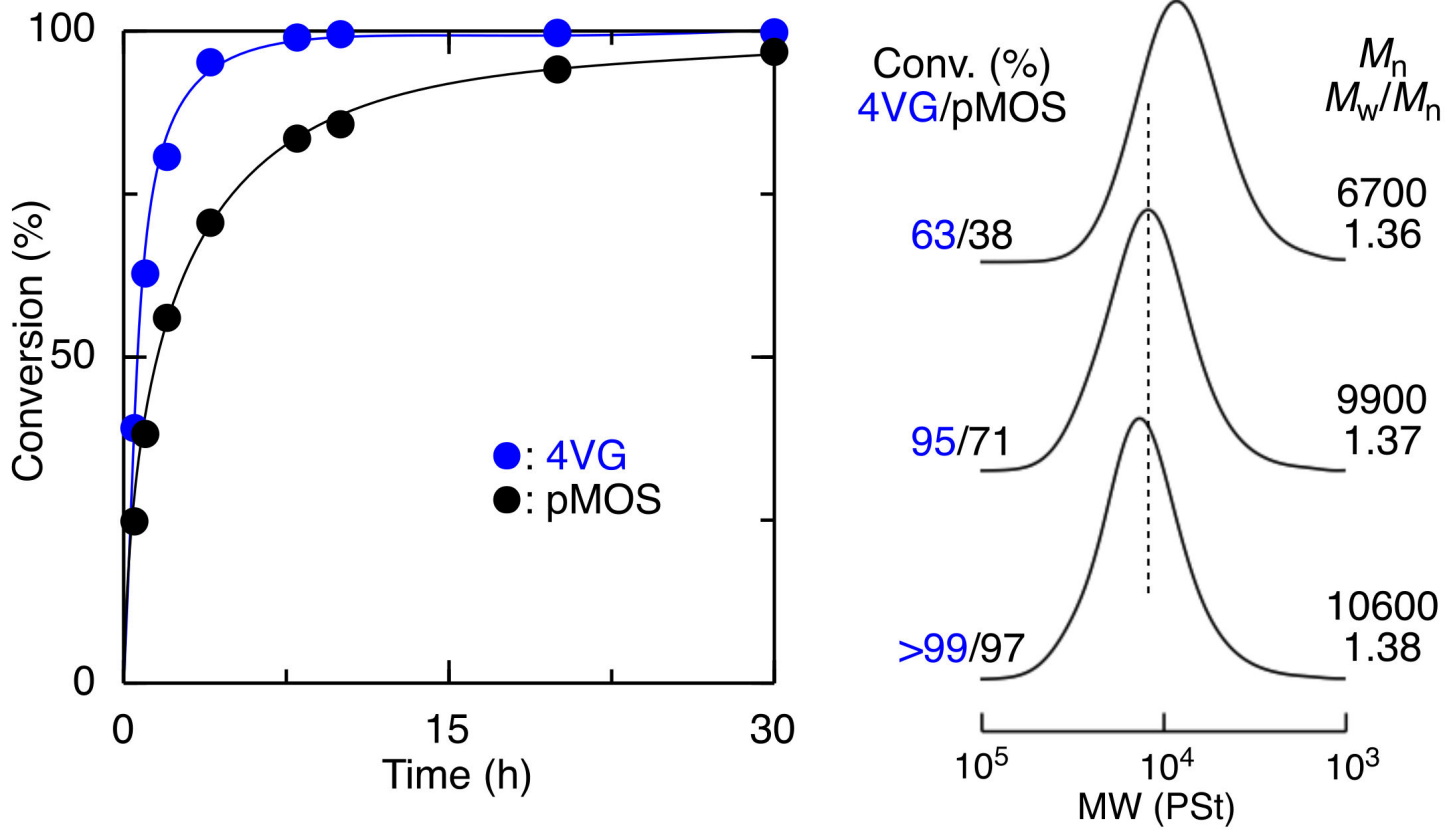
Statistical controlled/living cationic copolymerization of 4VG and pMOS with **1**/BF_3_·OEt_2_ in CH_3_CN/CH_2_Cl_2_ (8/2) at 0 °C; [4VG]_0_/[pMOS]_0_/[**1**]_0_/[BF_3_·OEt_2_]_0_/[H_2_O]_0_ = 100/100/4.0/2.0/200 mM.

The statistical random copolymerization of 4VG and pMOS was first conducted under the same conditions as for the homopolymerization except for the use of a mixture solvent of CH_3_CN and CH_2_Cl_2_ due to the solubility of the resulting polymer. The **1**/BF_3_·OEt_2_-based system was applied for the 4VG/pMOS copolymerization at 0 °C in which the comonomer feed ratio was 1:1 (Figure 4). Both of the monomers were consumed quantitatively after 30 h and 4VG reacted slightly faster than pMOS probably due to higher number of electron-donating substituents on the aromatic ring of 4VG. The SEC curves of the obtained copolymer also shifted to the higher molecular-weight region with relatively narrow MWDs (*M*_w_/*M*_n_ ~ 1.3). The formation of the controlled/living copolymer was also confirmed by ^1^H NMR and MALDI-TOF MS (Figure S1). In particular, the MALDI-TOF-MS spectrum showed complicated peaks that represent the copolymers of 4VG and pMOS units distributed statistically, and the spectrum is free of the peaks of the corresponding homopolymers as in the case of pHS.

**Figure 5:**
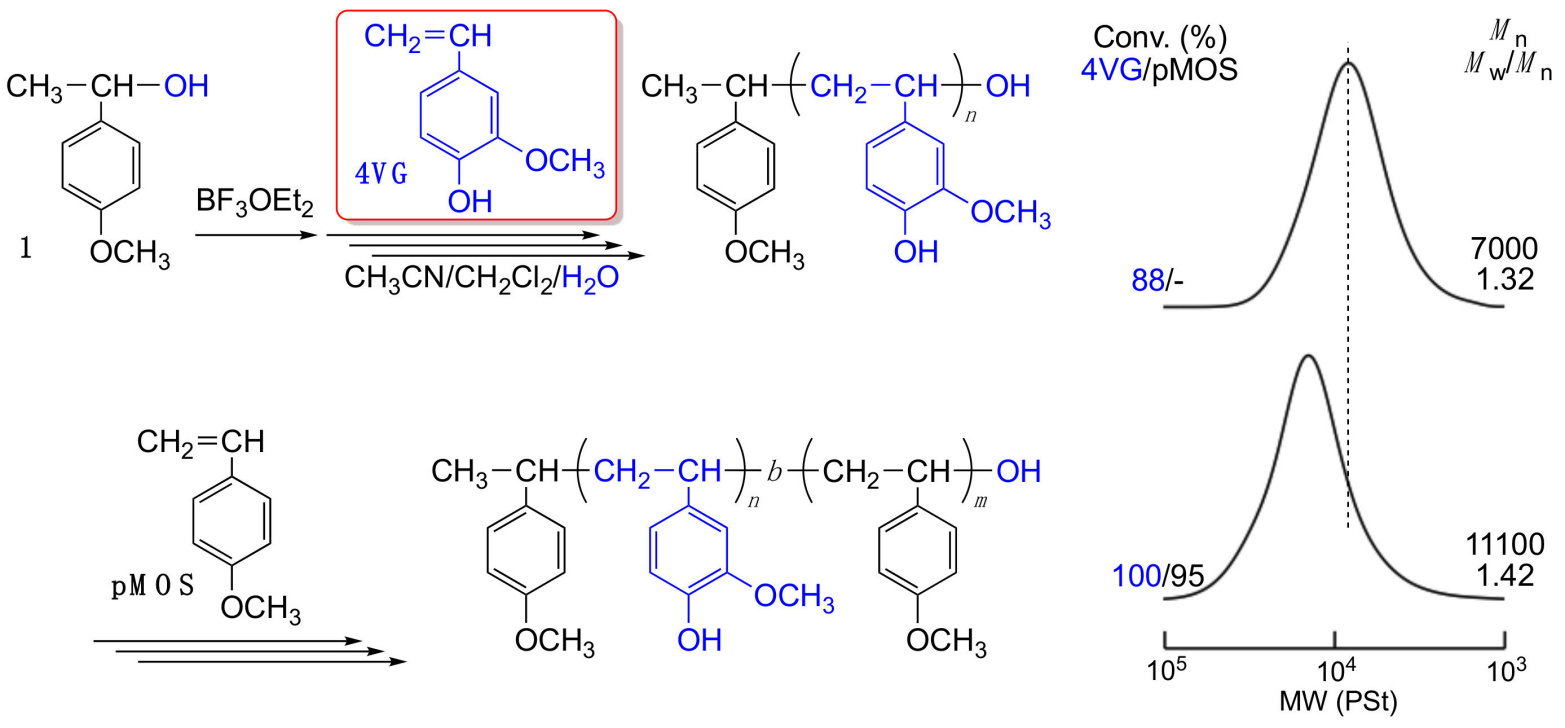
Block copolymerization of 4VG and pMOS with **1**/BF_3_·OEt_2_ in CH_3_CN/CH_2_Cl_2_ (8/2) at 0 °C; [4VG]_0_/[pMOS]_add_/[**1**]_0_/[BF_3_·OEt_2_]_0_/[H_2_O]_0_ = 100/100/4.0/2.0/200 mM.

Furthermore, the sequential block copolymerization of 4VG and pMOS was also investigated using the BF_3_·OEt_2_-based initiating system. The homopolymerization of 4VG was first performed with R–OH/BF_3_·OEt_2_ in the presence of water at 0 °C in the mixed solvent of CH_3_CN and CH_2_Cl_2_. After polymerization of 4VG at ca. 90% conversion, pMOS as the second monomer was added to the reaction mixture. The second polymerization also proceeded well even after monomer addition. After the second polymerization, the SEC curves of the obtained copolymer also shifted maintaining relatively narrow MWDs, indicating the formation of the block copolymer (Figure 5). The block copolymer was also confirmed by ^1^H NMR, where the incorporated ratio of the two monomers was in good agreement with the calculated values (Figure S2). Thus, the BF_3_·OEt_2_-based aqueous-cationic system was also effective for controlling statistical/random and sequential block copolymerization of 4VG and pMOS to form a copolymer with hydrophilic and more hydrophobic segments.

### 3.3. Properties of the Naturally-Derived Phenolic Polymers from Vinylguaiacol

**Figure 6:**
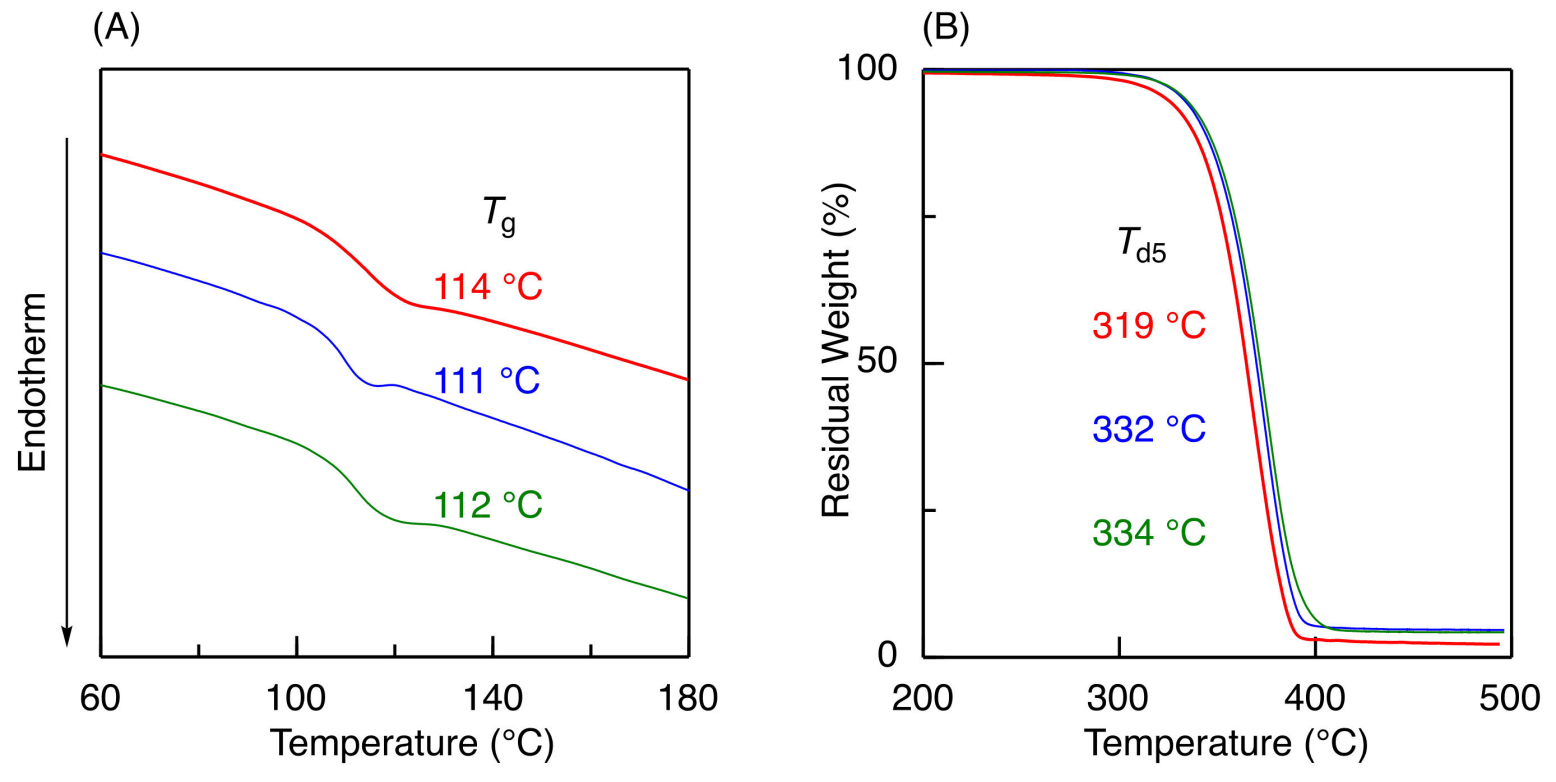
Differential scanning calorimetry (**A**) and thermogravimetric analysis (**B**) for poly(4VG) [*M*_n_ (SEC) = 11,800, *M*_w_/*M*_n_ = 1.28] (red line), poly(4VG-*stat*-pMOS) [*M*_n_ (SEC) = 10,600, *M*_w_/*M*_n_ = 1.38, 4VG/pMOS = 51/49] (blue line), and poly(4VG-*block*-pMOS) [*M*_n_ (SEC) = 11,100, *M*_w_/*M*_n_ = 1.42, 4VG/pMOS = 51/49] (green line).

The thermal properties of the 4VG-based phenolic polymers were then evaluated by differential scanning calorimetry (DSC) and thermogravimetric analyses (TGA) (Figure 6). Figure 6A shows the DSC profiles of the homopolymer and copolymer of 4VG. The glass transition temperature (*T*_g_) of the 4VG homopolymer that has a molecular weight of approximately 10 K was 114 °C, which is almost the same value as that of poly(4VG) obtained via radical polymerization of the protected-4VG and deprotection process. Upon copolymerization with pMOS, the *T*_g_ values did not change considerably, where the *T*_g_ values for the statistical and block copolymers were 111 and 112 °C, respectively. This is because the homopolymer of pMOS has almost the same *T*_g_ as poly(4VG). However, the copolymerization with nonfunctionalized pMOS slightly improved the thermal degradation temperature by TGA, as shown in Figure 6B. The thermal degradation of poly(4VG) began below 300 °C, of which the 5% weight-loss temperature was 319 °C; however, those of the statistical and block copolymers were higher than 330 °C.

To check the amphiphilic nature of the homopolymer and copolymer of 4VG, the solubilities of the phenolic copolymers were also evaluated in various solvents (Table 1). As previously reported, the amphiphilic poly(4VG) was easily soluble in methanol and aqueous sodium hydroxide (1.0 M) solution as well as in acetone, THF, and ethyl acetate (EtOAc) but insoluble in toluene and chloroform. The solubility to an aqueous alkali solution is similar to poly(vinylphenol), which is widely used industrially as a photoresist material. Regarding the solubility of the copolymers with pMOS, they were more easily dissolved in chloroform but less soluble in methanol due to the introduction of more hydrophobic pMOS units. However, the random/statistical copolymer of 4VG and pMOS exhibited solubility in aqueous NaOH solution at a low concentration (0.2 wt %). Thus, the amphiphilic nature of the 4VG-based phenolic polymer could be tuned via copolymerization with more hydrophobic pMOS.

## 4. Conclusions

In this study, we reported the water-based direct-controlled/living cationic polymerization of a plant-derived phenolic styrene, 4VG, to develop novel naturally-derived amphiphilic polystyrenes. 4VG was successfully and quantitatively transformed into novel bio-based polymers by the R–OH/BF_3_·OEt_2_ system without using any protective groups for the phenolic functional groups. In addition, 4VG could be copolymerized with a petroleum-derived styrene derivative, pMOS, in a controlled fashion to afford well-defined statistical and block copolymers with amphiphilic properties. We believe that this polymer can be expected not only to be used as a novel styrenic bio-based polymer but also as a material with amphiphilic nature for some applications, such as for photoresist and biomedical applications, due to its unique solubility and potential anti-oxidizing ability.

## Figures and Tables

**Table 1:** Solubility of various phenolic polystyrenes from 4VG *^a^.*

Polymer *^b^*	H_2_O	NaOH aq	CH_3_OH	Acetone	THF	EtOAc	CHCl_3_	Toluene
Poly(4VG)	−	++	++	++	++	++	−	−
Poly(pMOS)	−	−	−	++	++	++	++	++
Poly(4VG-*stat*-pMOS)	−	+	−	++	++	++	++	−
Poly(4VG-*block*-pMOS)	−	−	−	++	++	++	++	−

*^a^* “++”: soluble (1 wt %), “+”: soluble (0.2 wt %), “–”: insoluble. *^b^* The polymer samples are the same as in Figure 6.

## Supplementary Materials

The following are available online at http://www.mdpi.com/2073-4360/10/12/1404/s1, Figure S1: ^1^H NMR and MALDI-TOF-MS spectra of the statistical random copolymer of 4VG and pMOS obtained with **1**/BF_3_·OEt_2_, Figure S2: ^1^H NMR spectra of poly(4VG) and 4VG-*b*-pMOS block copolymer obtained with **1**/BF_3_·OEt_2_.
