# Supplementary material for: Naturally-Derived Amphiphilic Polystyrenes Prepared by Aqueous Controlled/Living Cationic Polymerization and Copolymerization of Vinylguaiacol with R–OH/BF3·OEt2
Source: Polymers (Basel). 2018 Dec 18;10(12):1404. doi: 10.3390/polym10121404 (PMC6401896; doi:10.3390/polym10121404)
Supplement: Supplementary file 1 [file polymers-10-01404-s001.pdf]

Supporting Information

Naturally-Derived Amphiphilic Polystyrenes Prepared by Aqueous Controlled/Living Cationic Polymerization and Copolymerization of Vinylguaiacol with R–OH/BF_3·OEt_2

Hisaaki Takeshima, Kotaro Satoh* and Masami Kamigaito*

Department of Molecular and Macromolecular Chemistry, Graduate School of Engineering, Nagoya University, Furo-cho, Chikusa-ku, Nagoya 464-8603, Japan

E-mail: satoh@chembio.nagoya-u.ac.jp, kamigait@chembio.nagoya-u.ac.jp

Contents:

Figure S1······························ S2

Figure S2······························ S3

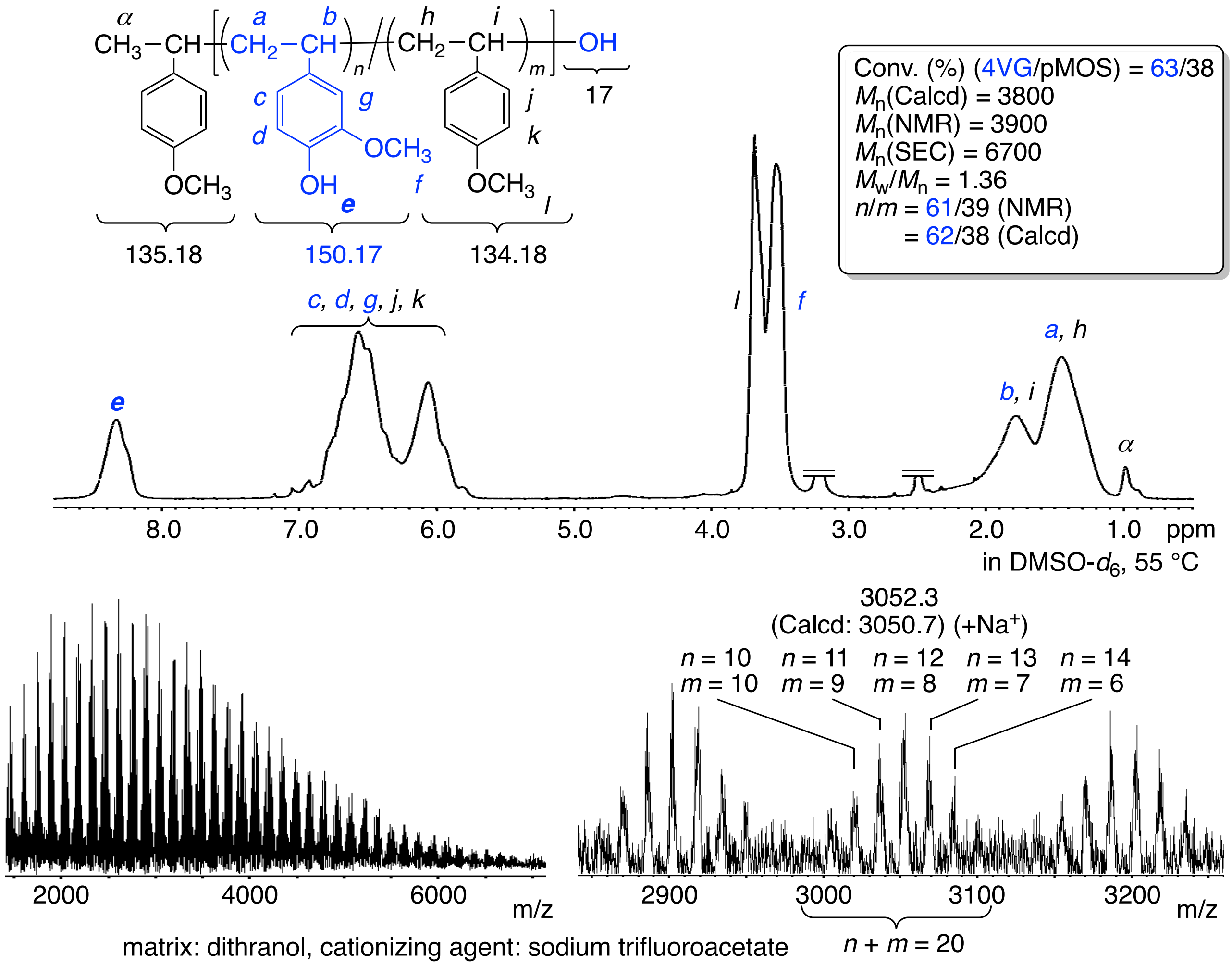

Figure S1. ^{1}H NMR and MALDI-TOF-MS spectra of the statistical random copolymer of 4VG and pMOS obtained with **1**/$BF_3 \cdot OEt_2$ (the same experiments as for Figure 4 in the main text): $[4VG]_0/[pMOS]_0/[\mathbf{1}]_0/[BF_3 \cdot OEt_2]_0/[H_2O]_0$ = 100/100/4.0/2.0/200 mM in CH_3CN/CH_2Cl_2 (8/2) at 0 °C.

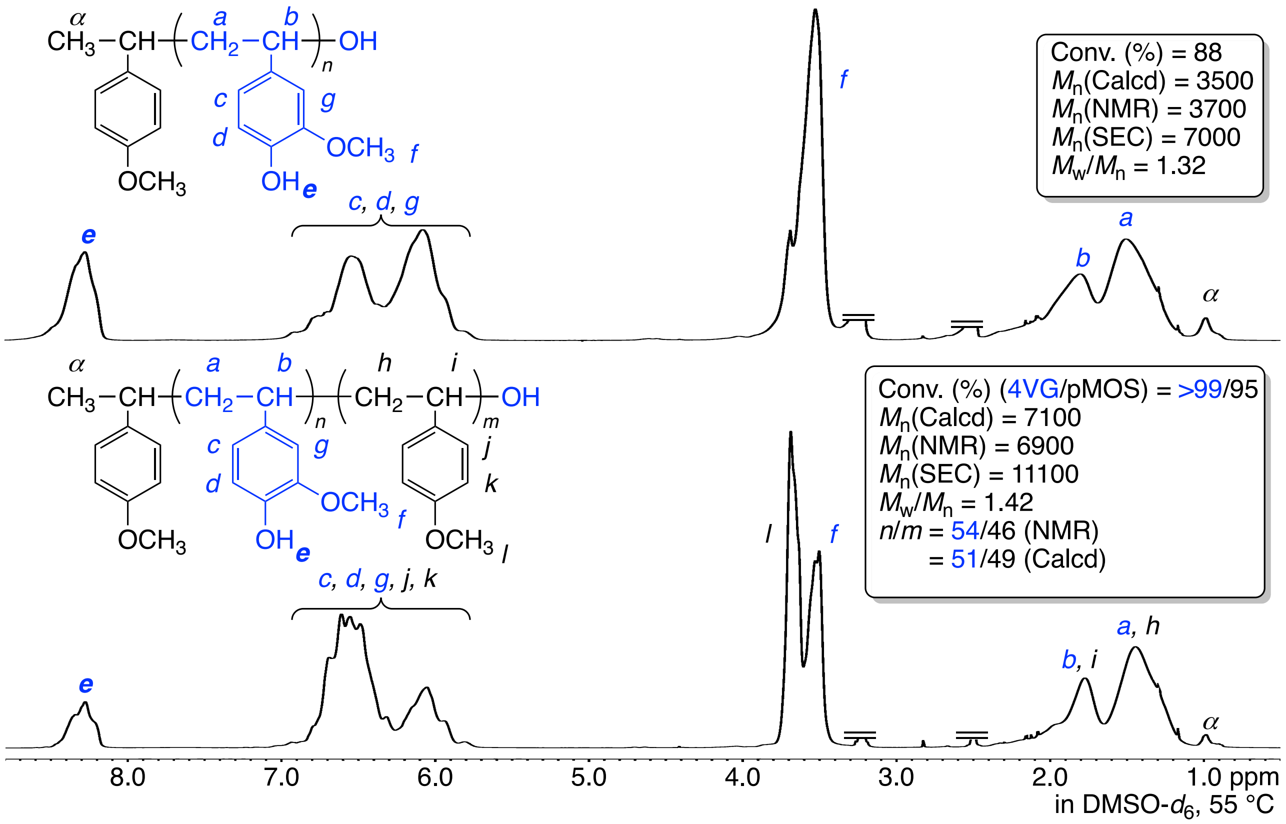

Figure S2. ^{1}H NMR spectra of poly(4VG) and 4VG-*b*-pMOS block copolymer obtained with **1**/BF_3·OEt_2 (the same experiments as for Figure 5 in the main text): $[4VG]_0/[pMOS]_{add}/[\mathbf{1}]_0/[BF_3{\cdot}OEt_2]_0/[H_2O]_0$ = 100/100/4.0/2.0/200 mM in CH_3CN/CH_2Cl_2 (8/2) at 0 °C.
